# Zooarchaeology of the Modern Era: An Introduction

**DOI:** 10.1007/s10761-022-00670-7

**Published:** 2022-08-06

**Authors:** Eric Tourigny, Rebecca Gordon

**Affiliations:** 1 School of History, Classics and Archaeology, Newcastle University, Newcastle upon Tyne, NE1 7RU UK; 2 Bluebell Close, Bedford, MK42 0RN UK

**Keywords:** Zooarchaeology, Human-animal relationships, Social zooarchaeology, Modern era

## Abstract

The last 500 years is characterized by immense socioeconomic and environmental transformations on a global scale. Animals were significantly affected by these processes but were also central to many of the transformations that shaped the modern world. While there has been a growing number of researchers investigating animal bones from archaeological sites from this period, the “Zooarchaeology of the Modern Era” working group provides the first dedicated forum for these scholars to meet. This paper introduces a special collection of studies which resulted from the first meeting of this research group and explores how these investigations help us understand our modern world.

## Introduction

This collection of papers emerged from the first official meeting of the “Zooarchaeology of the Modern Era Working Group." The working group has been recognized formally by the International Council for Archaeozoology (ICAZ) and the meeting was held virtually on December 4, 2020. Despite the Covid-19 pandemic, the meeting was attended by over 108 participants from 26 countries, thus demonstrating the global reach and enthusiasm of zooarchaeologists studying our recent past.

Over the past few decades, many zooarchaeologists have turned their attention to “recent,” “historical,” “post-medieval” or “modern” faunal assemblages, recognizing the importance of this period in understanding the world in which we live (for reviews, see Cruz 2020; Landon 2005, 2009; Landon and Opishinski 2020; Thomas and Fothergill 2014). While some of these scholars have occasional opportunities to meet and present their research through one-off sessions within conferences organized by societies dedicated to the archaeology of later periods (e.g., annual meetings of the Society for Historical Archaeology or The Society for Post-Medieval Archaeology), such venues do not provide regular occasions to discuss zooarchaeological issues and are often inaccessible to individuals living in certain parts of the world. This new working group represents a dedicated space for an emergent global community of scholars to meet, regardless of where they are based or where they conduct their research. The working group “aims to connect researchers, encourage collaborations, intellectual exchange, and promote future research within the discipline” (ICAZ 2018) and plans to meet regularly, either as part ICAZ’s quadrennial conference or through dedicated hybrid (in person and online) working group meetings held in intervening years.

## Defining the “Modern Era”

The working group defines the Modern era as representing the last ~ 500 years, including what is often referred to as the postmodern age (from ca. 1500CE to present). We recognize that the term “modern” is not used by all archaeologists and historians to refer to the last 500 years; but other terminology can be Eurocentric and exclusionary. In Europe, the term “post-medieval” is used to define this time span, but this term is inapplicable to parts of the world without a “medieval” period (Gilchrist 2005; Orser 2004:8). The term “historical archaeology” is popular in the Americas where it was first applied to distinguish between periods where written texts were available alongside archaeological evidence to interpret the past (Orser 2004:9). However, application of this term inappropriately suggests earlier periods are ahistorical and can lead to a coloniser/colonized dichotomy (Beaudoin 2019:19–21). There are also difficulties in applying the definition of historical archaeology as a text-aided subdiscipline to parts of the world where written histories predate the last 500 years (Gilchrist 2005:330).

“Historical zooarchaeology” is commonly deployed in the Americas and elsewhere (Cruz 2020; Landon 2009; Landon and Opishinski 2020), but these often focus on parts of the world colonized by Europeans. When forming this working group, we wanted to be inclusive of regions outside of Europe including those without strong links to European colonial projects – thus avoiding the hegemony of European timelines that have potentially limited participation in discussions (Tourigny et al. 2020:371). We equally recognize that, while many researchers studying zooarchaeological assemblages from the past 500 years can and do make use of documentary sources, not all can or need to. Similarly, while many studies do discuss culture contact and impacts of colonisation, not all research within the past 500 years need touch on these topics. What does link this research is that the people were living in an increasingly connected world, allowing archaeologists to focus on a range of different themes to explore these interactions. Whether research is focused on one culture group in one location or contact between groups in multiple locations, the data and methods have value to all researching this period.

This working group adopts a broad definition of the term “modern,” referring to a period in global history that experienced large socioeconomic and environmental transformations – from shifts in the distribution of wealth to the rise of consumerism, industrialization and globalization to climate change. We recognize that animals played a fundamental role in the events that shaped our modern world and our research group is dedicated to studying these shared themes of human-animal interaction in this period. These last 500 years witnessed the movements and introductions of species on great scales, from rural to urban areas and across continents. The period also witnessed intensified breeding and the industrialization of the food chain, resulting in the emergence of changed population demographics and new health issues for people and animals (e.g., Fothergill et al. 2012; Thomas et al. 2014), impacted local and global environments (see Deagan 1996), led to the development of new foodways, and changed the ways people relate to the animals in their lives (e.g., Thomas 2005; Puputti 2008).

## Practicing Zooarchaeology of the Modern Era

Comprehensive reviews of zooarchaeological studies covering this time period have provided excellent syntheses of the current state of research in the Americas (Cruz 2020; Landon 2005, 2009; Landon and Opishinski 2020) and in Europe (e.g., Broderick 2014; Thomas and Fothergill 2014). These highlight how research is united by a shared interest in specific themes and the application of specific methods. Most studies of this period include some level of assessment of diet and foodways, allowing archaeologists to investigate culture change and identity through food consumption (e.g., Franklin 2001; Gifford-Gonzalez and Sunseri 2007; Salmi et al. 2014). Studies seeking to reconstruct animal husbandry practices have also been popular, and continue to provide information on landscape change, the development of economies, and livestock breeding more generally (e.g., Cosette and Horard-Herbin 2003; Davies and Gavey 2013). As new analytical techniques have developed and/or matured over the past two decades, zooarchaeological studies are increasingly finding new ways to address research questions and integrate multiple types of data (e.g., data derived from biomolecules, pathologies, metrics) (Landon and Opishinski 2020:578–579). Research is also being increasingly informed by social zooarchaeological perspectives (Russell 2012; Sykes 2014), with interpretations framed through a posthumanist lens (Ritvo 2007) that considers the full-range of human-animal relationships present in the archaeological record.

While the research landscape is looking positive, these reviews also identify challenges faced by those in our working group. Thomas and Fothergill (2014:13) point to the fact that archaeological deposits from the modern era are often overlooked by commercial archaeology firms who deem them not interesting enough. It is well documented that not all countries have heritage legislation that protect recent deposits (for examples, see Tourigny et al. 2018, 2020). Subsequently, zooarchaeologists can find themselves working with limited contextual information and datasets that are not as representative as they could be. Residuality, particularly in built up urban areas, can pose a challenge to later faunal assemblages which can be truncated by modern development resulting in contaminated contexts with material of a different date (Albarella 2016). Many face increasing challenge of where to store and curate materials, especially if these materials are deemed less important than older assemblages (e.g., Broderick 2014:27).

## This Issue

This collection of papers highlight some of these trends in zooarchaeological approaches to the modern era. Twiss (2012) and Peres (2017) have demonstrated how dietary reconstructions do not simply identify what people ate in the past –zooarchaeological analysis can contribute information about the cultural, environmental, and social context of the decisions that shaped consumption practices. Peres’ contribution to this series is a prime example of research that seeks to provide further context behind the development of local economies and identities in a changing world. The paper investigates how Spanish mission communities in seventeenth-century Florida incorporated animals into their foodways to varying degrees of success and how some of these animals became profitable commodities. Arias et al. similarly demonstrate the importance of identifying the cultural meaning behind food consumption practice as they examine the earliest evidence of changes in meat consumption following the earliest interactions between Indigenous people and Europeans in the south-central Andes.

Siddiq’s analysis permits the reconstruction of the foodways of elite Ottoman soldiers while also getting a sense of local pastoral and hunting practices. Their paper demonstrates the importance of linking knowledge gained from the analysis of single deposits to a broader context. They further emphasize the importance of using zooarchaeological data to understand a range of human-animal relationships, beyond food consumption, in under-investigated periods.

Yeoman’s and Quinlan’s contributions engage explicitly with social zooarchaeology, considering the important relationships people formed with animals beyond economic services or dietary contributions. Yeomans’ paper explores a global practice that leaves little evidence in the zooarchaeological record. Through a combination of archaeological evidence from southeast Arabia and ethnographic data from around the world, they explore the unique practice of fishing with remora while also discussing the complex relationships that developed between humans and their non-human fishing partners.

Quinlan’s paper builds on the recent use of animal osteobiographies (Hull 2020; Tourigny et al. 2016) to better understand the relationship between humans and animals while giving equal weight to the animal’s perspective. Osteobiographies allow the zooarchaeologist to construct full life (and death) history narratives as opposed to relying on the interpretation of large datasets obtained through quantitative analyses (Hosek and Robb 2019). In their paper, Quinlan examines the life and death of a young dog buried below an eighteenth-century tavern.

Landon and Opishinski (2020:578) identify a key challenge as the integration of new analytical techniques (e.g., biomolecular studies) within zooarchaeological practice and our ability to apply “humanistic and theoretically sophisticated interpretations that foreground human action and agency.” In their contribution, Kennedy and Guiry combine stable isotope and zooarchaeological data to explore the sourcing and transportation of meat in the nineteenth-century American West, while highlighting the impact Chinese culture had on the transcontinental railroad. In doing so, they highlight the ability to investigate diaspora communities using biomolecular approaches.

Two other papers combine disparate datasets with more traditional zooarchaeological analyses. Heinrich and Gall’s contribution uses geographic information systems (GIS) to investigate the complex intersection of meat consumption, social class and cultural affinity of enslaved, free black and white communities in eighteenth- and nineteenth-century Delware, USA. Their research demonstrates how zooarchaeologists can better visualize data to reveal changing consumption patterns across time and space. In the final contribution, Welker and colleagues adopt morphometric analyses to demonstrate new ways zooarchaeologists can investigate the introduction, spread, and breeding of domesticated species. With the example of the chicken, they use these data to consider implications of changing body size on human-chicken relationships over time.

## Conclusion

Working with assemblages from the recent past can provide a better understanding of the world in which we live and the development of our current relationships with animals. Through the investigation of abundant archaeological and documentary sources, parallels can be drawn to further contribute to our understanding of animal-human relationships in the deeper, poorly documented past. The Zooarchaeology of the Modern Era Working Group will continue to meet regularly, providing a venue for the global research community to meet and exchange ideas and experiences, share their knowledge, encourage collaboration, and promote future research within the discipline. We encourage all zooarchaeologists engaged or interested in the last 500 years of history to take part in future activities, particularly those investigating geographical regions and/or cultural groups that are underrepresented in the current literature. As others have observed before, there is great potential in the zooarchaeology of this period as many themes remain underexplored, interdisciplinary collaborations are increasingly becoming the norm, and further innovations in research approaches and technologies are on the horizon (Landon 2009; Thomas and Fothergill 2014).
